# COVID-19 Pneumonia: ST-segment Elevation Myocardial Infarction (STEMI) and Myocarditis

**DOI:** 10.7759/cureus.11901

**Published:** 2020-12-04

**Authors:** Azhar Ali, Mohammad Fawad Khattak, Muhammad W Khan

**Affiliations:** 1 Cardiology, Russell Hall Hospital, Dudley, GBR

**Keywords:** coronary artery thrombosis, st-elevation myocardial infarction (stemi), covid 19, covid-induced myocarditis

## Abstract

Severe acute respiratory syndrome coronavirus 2 (SARS-CoV-2) is associated with numerous cardiac complications. We describe a case of type 1 ST-segment elevation myocardial infarction (STEMI) with focal myocarditis unmasked in a patient infected with SARS-CoV-2 with no previous cardiac history, in the hope of increasing understanding of the severity and possible complications of coronavirus disease 2019 (COVID-19) and improving its clinical management.

## Introduction

Thromboembolism is a common and serious complication of coronavirus disease 2019 (COVID-19). Its incidence increases with the severity of the disease. Factors like prolonged bed stay, excessive inflammation, and hypoxia may be contributing factors [1]. Numerous case reports have emerged detailing the cardiovascular complications of COVID-19 and there are different hypotheses regarding the pathogenesis of these.

## Case presentation

fA 59-year-old gentleman presented to the accident and emergency department with a ten-day history of productive cough, fever, rigors, and anosmia. His past medical history consisted of diet-controlled diabetes mellitus; he was an ex-smoker with six pack-years, but he was otherwise fit and well.

His temperature recorded was 38.9°C. He was tachypnoeic and had tachycardia of 104 beats per minute. SpO2 was 90% on 15L of oxygen. There were bi basal crackles on chest examination. Rest of the systemic examination was normal.

Arterial blood gasses (ABGs) taken on 15L of oxygen was showing type 1 respiratory failure. Inflammatory markers were raised with associated lymphopenia. His blood results from admission are reported in Table 1.

**Table 1:** Blood results from admission with troponin trend since onset of the chest pain WBC: white blood cells; CRP: C-reactive protein; CK: creatine kinase; eGFR: estimated glomerular filtration rate; LDH: lactate dehydrogenase.

	On admission	3rd day	On discharge
WBC	12.7 × 10^9 ^/L	15 × 10^9 ^/L	
CRP (mg/L)	205	310	12
Lactate(mmol/L)	1.6		
Lymphocytes	0.31 × 10^9 ^/L		
CK (U/L)	190		
eGFR (ml/min)	40		77
LDH	0.452		
Creatinine (mg/dL)	161		95
D-dimer (ng/ml)	1200		

**Figure 1:**
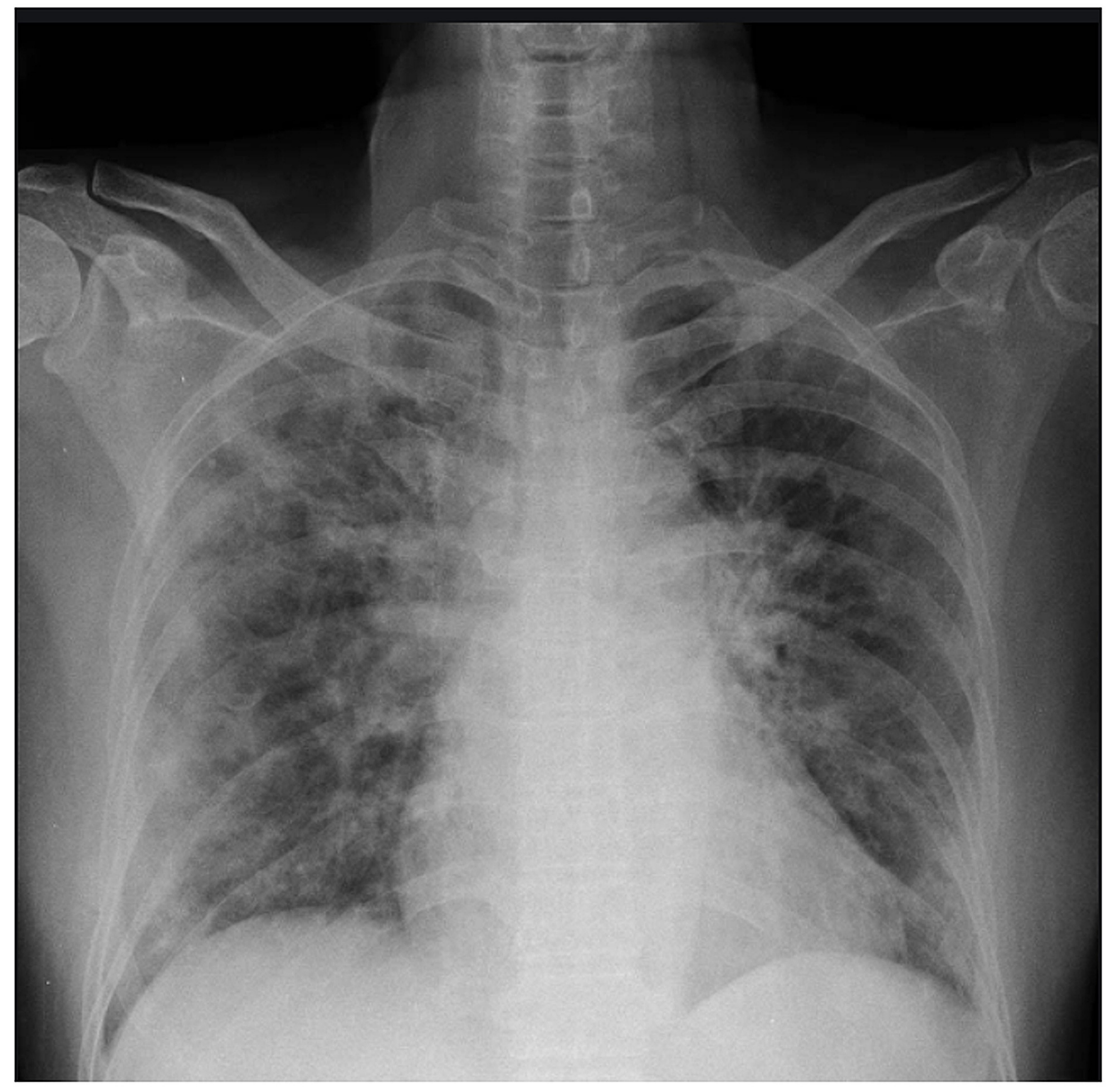
Chest X-ray showing bilateral ground glass opacities, typical for COVID-19

Chest X-ray showed bilateral ground glass opacities consistent with COVID-19 pneumonia (Figure 1).

The patient received prophylactic enoxaparin and he was kept on continuous positive airway pressure for two days on medical high dependency unit (HDU). He was successfully weaned off of continuous positive airway pressure (CPAP) on his second day of admission.

**Figure 2:**
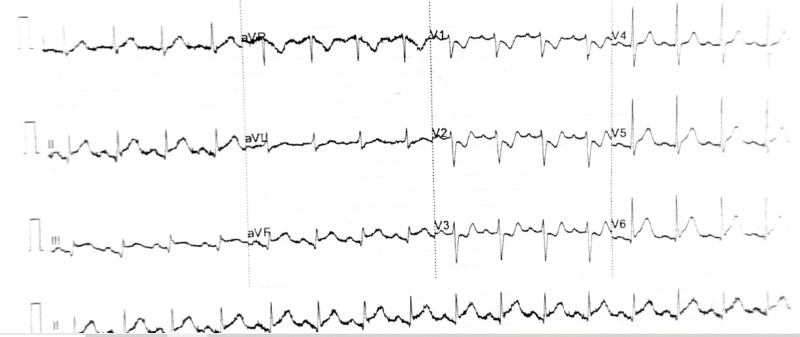
12-lead ECG of the patient taken during chest pain

On the third day of admission, the patient acutely developed retrosternal chest pain radiating to his neck and arm with ECG findings consistent with STEMI in the infero-posterior leads (Figure 2). Shortly the patient had pulsed ventricular tachycardia (VT) followed by primary ventricular fibrillation (VF) arrest which was successfully cardioverted with a single unsynchronized 360 J. He had further episodes of pulseless VT for which a cycle of cardiopulmonary resuscitation (CPR) was given before return of spontaneous circulation (ROSC).

He was loaded with aspirin 300 mg, clopidogrel 300 mg, and successfully thrombolysed with IV alteplase, after discussing with an interventional cardiologist who deemed not for transfer for primary percutaneous coronary intervention (PCI). It was followed by a 24-hour unfractionated heparin infusion.

**Figure 3:**
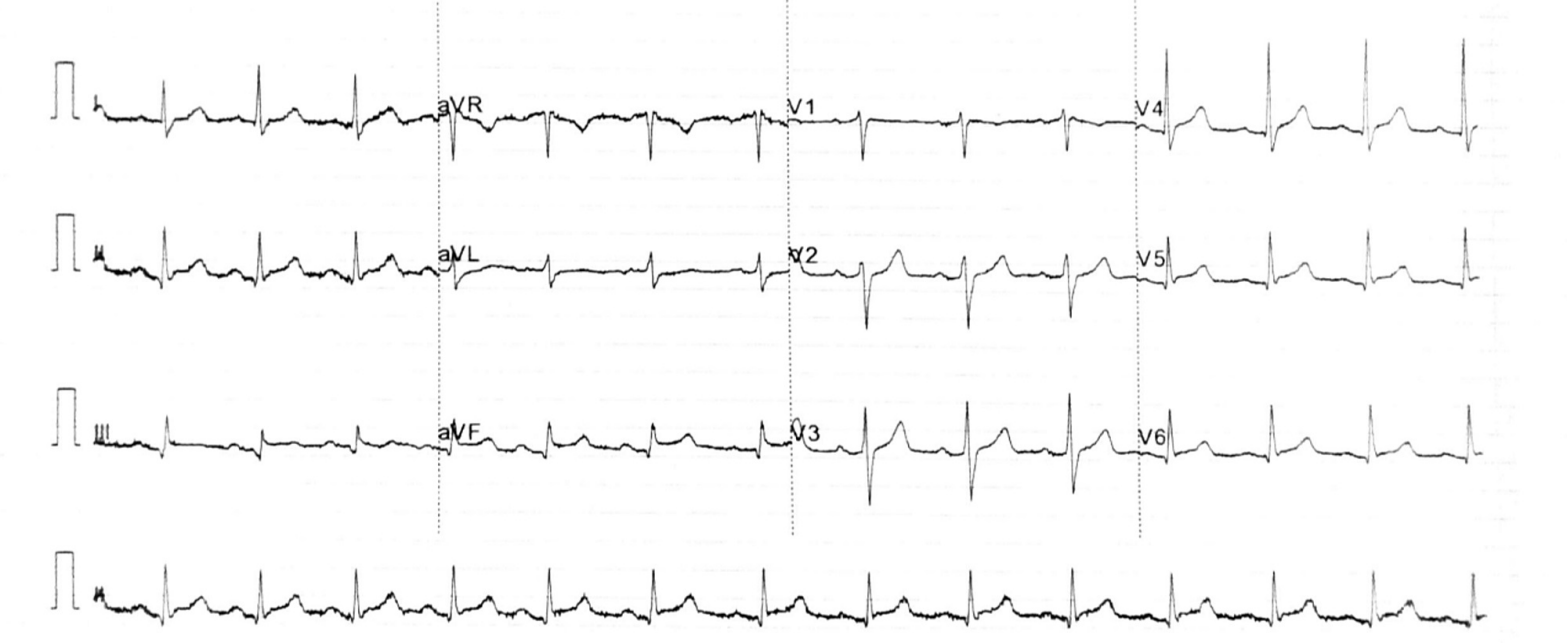
Post thrombolysis 12-lead ECG

Bedside echocardiogram revealed severe left ventricular (LV) systolic dysfunction (left ventricular ejection fraction (LVEF) 32%) with hypokinetic inferior and lateral walls. His troponin was 482 ng/L which peaked up to 584 ng/L after six hours of chest pain. Post thrombolysis ECG showed more than 50% reduction in ST-segment elevation (Figure 3). Also, the patient improved clinically. His blood tests were improving and the echocardiogram on discharge showed good bi-ventricular systolic function with no significant regional wall motion abnormality.

Outpatient CT coronary angiogram revealed flow-limiting diffuse severe three-vessel coronary disease with very high plaque burden. Coronary Artery Disease-Reporting and Data System (CAD-RADS) was 4b.

Subsequent cardiac MRI after two weeks of discharge confirmed focal mid inferolateral LV infarct which was non-viable. LV systolic function was mildly impaired in the presence of regional wall motion abnormality. Also, there was evidence of LV apical lateral wall myocarditis.

The patient was referred for invasive coronary angiography and consideration of coronary artery bypass graft surgery.

## Discussion

COVID-19 mainly affecting the respiratory system has been reported to have an association with both pre-existing and newly developed cardiovascular diseases, which worsen both its prognosis and mortality. Acute cardiac injury occurs in approximately 8%-12% of all patients increasing its mortality up to 51.2% among the hospitalized group, especially in older people with pre-existing other comorbidities [2].

Acute myocarditis, a potentially life-threatening disease was reported in up to 7% of COVID-19 related deaths [3]. Although its exact mechanism is unclear, myocardium damage could be related to cytokine storm from excessive inflammation or it could be secondary to direct viral involvement through angiotensin-converting enzyme 2 (ACE2) receptors which are highly expressed in the heart and lung [4].

Similarly, COVID-19 is a hypercoagulable state. The exact pathophysiology leading to the thromboembolic nature of the disease is still not clear. It is has been hypothesised that there could be activation of the thrombotic pathway by cytokines as part of the inflammatory process seen in COVID-19. Also, there is damage to the endothelial cells which causes its dysfunction and activation for thrombosis. Studies have shown that thromboembolic risk increases with disease severity and hospital stay. People with pre-existing cardiovascular diseases are at more risk of severe infection. Severe inflammation may cause atherosclerotic plaque disruption resulting in acute coronary syndromes [5].

## Conclusions

Myocarditis can mimic acute coronary syndrome. Myocarditis has more common association with COVID-19 and its high mortality makes it challenging to differentiate it from acute coronary syndrome. It is very important to make a correct diagnosis for prompt treatment. However endocardial biopsy is still the gold standard for the diagnosis of myocarditis but its invasive character and limited sensitivity restrict its generalized application. Echocardiography and cardiac serum biomarkers are easily available but lack enough specificity to differentiate reliably between the two entities. Cardiac magnetic resonance is useful not only to differentiate acute myocarditis from an ischemic event but also to identify alternative etiologies.
